# A rapid high-throughput sequencing-based approach for the identification of unknown bacterial pathogens in whole blood

**DOI:** 10.2144/fsoa-2020-0013

**Published:** 2020-04-29

**Authors:** Ofir Israeli, Efi Makdasi, Inbar Cohen-Gihon, Anat Zvi, Shirley Lazar, Ohad Shifman, Haim Levy, David Gur, Orly Laskar, Adi Beth-Din

**Affiliations:** 1 Department of Biochemistry & Molecular Genetics, Israel Institute for Biological Research, Ness Ziona, Israel; 2 Department of Infectious Diseases, Israel Institute for Biological Research, Ness Ziona, Israel

**Keywords:** *B. anthracis*, bacteremia, *F. tularensis*, high-throughput DNA sequencing, host/pathogen DNA ratio, whole blood samples, *Y. pestis*

## Abstract

High-throughput DNA sequencing (HTS) of pathogens in whole blood samples is hampered by the high host/pathogen nucleic acids ratio. We describe a novel and rapid bacterial enrichment procedure whose implementation is exemplified in simulated bacteremic human blood samples. The procedure involves depletion of the host DNA, rapid HTS and bioinformatic analyses. Following this procedure, *Y. pestis*, *F. tularensis* and *B. anthracis* spiked-in samples displayed an improved host/pathogen DNA ratio of 2.5–5.9 orders of magnitude, in samples with bacteria spiked-in at 10^3^–10^5^ CFU/ml. The procedure described in this study enables rapid and detailed metagenomic profiling of pathogens within 8–9 h, circumventing the challenges imposed by the high background present in the bacteremic blood and by the unknown nature of the sample.

## Lay abstract

Bloodstream infections are amid the major causes of human deaths among hospitalized patients. High-throughput DNA sequencing (HTS) of pathogens in whole blood samples has the potential to diagnose such infections, but this ability is hampered by the high host/pathogen nucleic acids ratio. We describe a novel and rapid bacterial enrichment procedure whose implementation is exemplified in simulated bacteremic human blood samples. Following this procedure, we were able to detect bacteria spiked-in at relevant concentrations within 8–9 h, circumventing the challenges imposed by the high background present in the bacteremic blood and by the potentially unknown nature of the sample.

Bacteremia that leads to sepsis and bloodstream infections are among ranked highly as causes of human death among hospitalized patients. Global evaluations of sepsis prevalence exceed 19 million cases per year. While only 2% of patients are admitted to the hospital with severe sepsis, they represent at least 10% of all intensive care unit admissions in the USA. Although major efforts are implemented in sepsis and bloodstream infections study, new therapeutic measures are scarce and mortality in patients with septic shock still remains extremely high [1–3]. Rapid diagnostics aiming at identification of bloodstream pathogens, can optimize the antimicrobial treatment regime, improve patient survival and decrease the financial impact of sepsis on healthcare systems worldwide [4].

Blood cultures remain the gold standard of sepsis diagnostics, however, this mode of diagnosis has several limitations: the test has a relatively long time frame (typically a few days) to run, producing false-negative results mainly due to growth dependence of the pathogen and the administration of empiric antibiotics [5,6]. Ultimately, early intervention by molecular detection of bacteria directly from whole blood could provide the most patient benefit and contribute to tailored antibiotic coverage of the patient early on in the course of the disease [4].

In the relatively simple scenario where there is a solid suspicion of a specific pathogen, molecular biology techniques, which are mostly PCR based, involving the amplification of a defined target, can be employed. These methods allow a rapid and straightforward identification compared with conventional culture-based methods, are highly sensitive and specific, and can yield results within 1 or 2 h, from only a few copies of the target genome in the sample [7,8]. However, PCR-based methods demand a certain level of prior information which is not always present. In the case of no previous information about the content of a sample (i.e., an unknown sample), or when there is a suspicion of an emerging or engineered new pathogen outbreaks, the ultimate nucleic acid-based detection method should be based on unbiased DNA sequencing. Sequencing of the entire content of a sample might provide not only an answer for the ‘yes or no question’ of whether a specific pathogen is present in the sample, but also answer the ‘what question', in other words, the question of what the sample contains, including the characterization of different genomic traits [9]. Such an approach is not dependent on the growth of organisms, and only the presence of their nucleic acid in the sample is needed for identification. Until a decade ago, the use of DNA sequencing to decipher the content of an unknown sample was not feasible. Traditional Sanger-based sequencing was not suitable for analyzing mixtures and was expensive, time-consuming and laborious. The emergence of high-throughput sequencing (HTS) technologies paved the way for the comprehensive detection of pathogens without any prior knowledge. These massively parallel sequencing platforms can sequence a heterogeneous mixture of genetic materials with high sensitivity and speed and with a lower cost per base compared with the traditional Sanger sequencing method [10,11]. HTS has other benefits apart from the improved detection of known and unknown pathogens in different samples; among these are the ability to detect nonculturable organisms and the ability to detect co-infections, drug resistance, antibiotic resistance and genetically modified or engineered organisms [12].

Direct diagnosis of whole blood samples utilizing HTS remains very challenging, owing to the variable and mostly low pathogen load, but especially due to a very high ratio of host/pathogen nucleic acids present (up to 10^8^:1 ratio of host DNA to pathogen DNA in blood samples). Therefore, the diagnosis of pathogens using HTS necessitates a host DNA depletion and/or pathogen enrichment step. A similar depletion strategy was employed for less challenging samples, in terms of host/pathogen ratio: cerebrospinal fluid and nasopharyngeal aspirate [13]. In this publication, the authors used few strategies, which ultimately demonstrated a 20- to 650-fold improvement of the host/pathogen ratio, an improvement ratio that might not be sufficient to assist diagnosis of bacteremia in direct whole blood samples. Additionally, in our hands, using similar methods as described in Hasan *et al.* [13] or a commercially available selective depletion of human DNA based on elevated methylation, did not improve the host/pathogen ratio in more than 100-fold [data not shown]. Recently, the use of the pathogen circulating cell-free DNA from plasma samples of septic patients by HTS has been used [14,15]. and demonstrated more sensitive and specific results than previous technologies. These promising studies used large HTS machines (i.e., Ilumina's HiSeq) which require a minimal sequencing time of 1 day, combining the total time needed from patient to answer to few days.

Very recently, Charalmpous *et al.* [16]. employed a new and promising depletion method, which combined saponin and the use of high salt active nuclease (SAN). This method demonstrates very promising results in direct HTS of pathogens derived from human sputum. The method is straightforward, relatively rapid and inexpensive. In our study, we combined our rapid HTS pipeline [17] with this depletion method and modified it to treat blood samples. We spiked-in whole blood samples with a variety of representative bacteria as a proof of concept for bacteremia diagnosis of unknown samples. Using our approach, we present, for the first time, the use of HTS for deciphering the genetic content of whole blood samples with unknown content, within a working day. This result is accomplished by a combination of the depletion step, a rapid library preparation procedure utilizing Nextera XT technology, generation of short reads (50–60 nt) with the MiSeq sequencer (Illumina) and prompt bioinformatics analyses conducted against all available bacterial, viral and fungal genomic sequences. Using this comprehensive and rapid approach, we were able to detect three CDC Tier 1 agents*: B. anthracis, F. tularensis and Y. pestis*, which acted as simulants of unknown samples, directly from whole blood samples, at relevant concentrations [18,19] and within a relatively short timeframe.

## Materials & methods

### Preparation of the bacterial spike-ins & human blood samples collection

*B. anthracis* Vollum ΔpXO1ΔpXO2 spores (ATCC 14578) were germinated in 200 μl of terrific broth for 2.5 h at 37°C. *Y. pestis* (EV76) was grown on brain–heart infusion agar (BHIA) plates (BD) for 48 h at 28°C. *F. tularensis holarctica* vaccine strain LVS was grown on cysteine heart agar (CHA) plates (GC medium base, supplemented with 1% [BD] and 1% hemoglobin (BBL) for 48 h at 37°C. Colonies were suspended in sterile phosphate-buffered saline (PBS) and CFU/ml was initially estimated by OD measurement. CFU counts were determined by plating 0.1 ml of serial dilutions that were made from the culture on BHIA enrichment plates, followed by 48-h incubation at 28°C, for *Y. Pestis*, 72-h incubation at 37°C for *F. tularensis* and 24-h incubation at 37°C for *B. anthracis*. *B. anthracis*, *Y. pestis* and *F. tularensis* (10^3^–10^5^ CFU/ml) were inoculated in 2 ml blood samples.

Human blood samples were obtained from the National Blood Services, MDA, Israel, under MDA research permit 08-0290. 2 ml of the blood samples were spiked-in with serial dilutions of *B. anthracis* strain Vollum ΔpXO1ΔpXO2 (ATCC 14578), *Y. pestis* strain EV76 [20] or *F. tularensis* LVS bacteria corresponding to 10^3^–10^5^ CFU/ml.

### Human DNA depletion & bacterial enrichment

Spiked-in blood samples (2 ml) were centrifuged at 300 ×*g* for 5 min, after which the supernatant (∼800 μl) was transferred to a new tube and centrifuged again at 14,000 ×*g* for 5 min. after which the supernatant was carefully removed and the pellet resuspended in 250 μl PBS. Next, we used saponin-based differential lysis method, that was adopted from [16] with slight modifications. Saponin (Sigma-Aldrich, MO, USA) was added to a final concentration of 2.2 % (250 μl of 4.4% saponin), mixed well and incubated at room temperature (RT) for 10 min to promote host cell lysis. Following this incubation, 350 μl of water was added and incubation was continued at RT for 30 s, after which 12 μl of 5 M NaCl was added to deliver an osmotic shock, lysing the damaged host cells. Samples were next centrifuged at 6000 ×*g* for 5 min, with the supernatant removed and the pellet resuspended in 100 μl of PBS. SAN buffer (5.5 M NaCl and 100 mM MgCl_2_ in nuclease-free water) was added (100 μl) with 10 μl SAN (Sigma-Aldrich) and incubated for 10 min at 37°C with shaking at 800 RPM for host DNA digestion. Finally, the host-DNA depleted samples were washed two-times with increasing volumes of PBS: 800 μl and then 1 ml. After each wash, the samples were centrifuged at 6000 ×*g* for 3 min, the supernatant discarded and the pellet resuspended in PBS. After the final wash, the pellet was re-suspended first in 100 μl PBS and then 100 μl ATL buffer (Qiagen, Hilden, Germany) and vortexed vigorously. The samples were then microwaved at maximum power for 7 min for sterilization and bacterial lysis and were used for DNA extraction.

### DNA extraction & quantification

DNA was extracted from the samples using DNA QIAamp DNA Blood Mini Kit (Qiagen) according to the blood and body fluids protocol in a QIAcube robot and was recovered in a 100 μl elution volume in ddH_2_O. The DNA was quantified using the Qubit fluorometer DNA HS Assay Kit (Invitrogen, CA, USA). The DNA was diluted to 0.25 ng/μl, as required by the Nextera XT sample preparation protocol.

### Sequencing library preparation & HTS

One nanogram (in 4 μl) of DNA was transferred to the library preparation step utilizing the Nextera XT DNA sample preparation kit (Illumina, CA, USA). The standard manufacturer protocol was modified to optimize library preparation from blood samples. For each sample, the 20 μl tagmentation reaction contained 5 μl of amplicon tagment mix (ATM), which includes the enzyme used for tagmentation, 10 μl of TD buffer, 1 ng (in 4 μl) of input DNA and 1 μl of 20 mg/ml bovine serum albumin (BSA). The tagmentation reactions were incubated in a thermal cycler at 50°C for 5 min. Subsequently, the tagmented DNA was amplified via limited-cycle PCR. The quality and quantity of the purified libraries were assessed using the high-sensitivity (HS) DNA kit on an Agilent TapeStation. The libraries were normalized to 3 nM, denatured with 0.2 N NaOH for 5 min and diluted 1:100 in HT1 buffer (Illumina) to a final concentration of 15 pM. The diluted libraries were sequenced with an Illumina MiSeq with 50 bp v2 run chemistry. The sequencing length was 50–60 nt in single-read mode. Each sample was spiked-in with 0.2 pM phiX174 library (Illumina). For the *F. tularensis* spike-in samples three different barcodes were used (the barcodes are included in the nextera XT library kit). On the Illumina MiSeq, the process of demultiplexing and generating the fastq data files required for downstream analysis is carried out automatically using the onboard PC.

### Bioinformatic analyses

All reads generated by HTS were taxonomically profiled with PathoScope 2.0 [21] using a constructed target genome library containing all complete bacterial (5414), viral (5777) and fungal (3451) genomes downloaded from NCBI (January 2018). The reads were aligned to these databases using the Bowtie2 algorithm [22] with the default PathoScope parameters. Using a limited database (compared with the whole NCBI database) facilitates the rapid bioinformatics analyses. The phiX174 sequence and similar sequences were manually curated from the results. Known HTS contaminants were also manually curated from the sequencing results and included the genera rhizobium/agrobacterium, sphingomonas, burkholderia, ralstonia, pseudomonas, stenotrophomonas and flavobacterium [23].

### Dual (bacterial specific & human) real-time PCR assays for *B. anthracis, Y. pestis* & *F. tularensis* & human DNA detection

Dual real-time PCR assays were performed in a 50 μl reaction volume using the SensiFAST™ Probe Lo-ROX kit (BIOLINE). Every mix contained bacterial specific primers and a FAM™ labeled probe and an x20 (2.5 μl) mix of the human *RNAseP* test, which contains primers with a Vic™ labeled probe (Applied Biosystems™). The PCR was carried out on a 7500 real-time PCR system (Applied Biosystems), under the following conditions: 2 min at 95°C followed by 40 cycles at 30 s 95°C and 30 s 60°C. The specific bacterial primers and probes are detailed below (primer F, primer R and probe): *B. anthracis* [24]: PL3_f (AAAGCTACAAACTCTGAAATTTGTAAATTG); PL3_r (CAACGATGATTGGAGATAGAGTATTCTTT); Tqpro_PL3 (6-FAM-AACAGTACGTTTCACTGGAGCAAAATCAA-BHQ-1). *Y. pestis* [25]: capF (GGATTACGATCTCTCGGATGTGA); capR (AGCCGGACAGACGAATAACTTC); Taq-CapR (6-FAM-TTGTGGCGACCTCTAACTCCATGAATATTCC-BHQ-1). *F. tularensis* [26] *(*ATCTAGCAGGTCAAGCAACAGGT); fopAR (GTCAACACTTGCTTGAACATTTCTAGATA); fopAP (6-FAM-CAAACTTAAGACCACCACCCACATCCCAA-BHQ-1).

## Results & discussion

**Figure 1.**
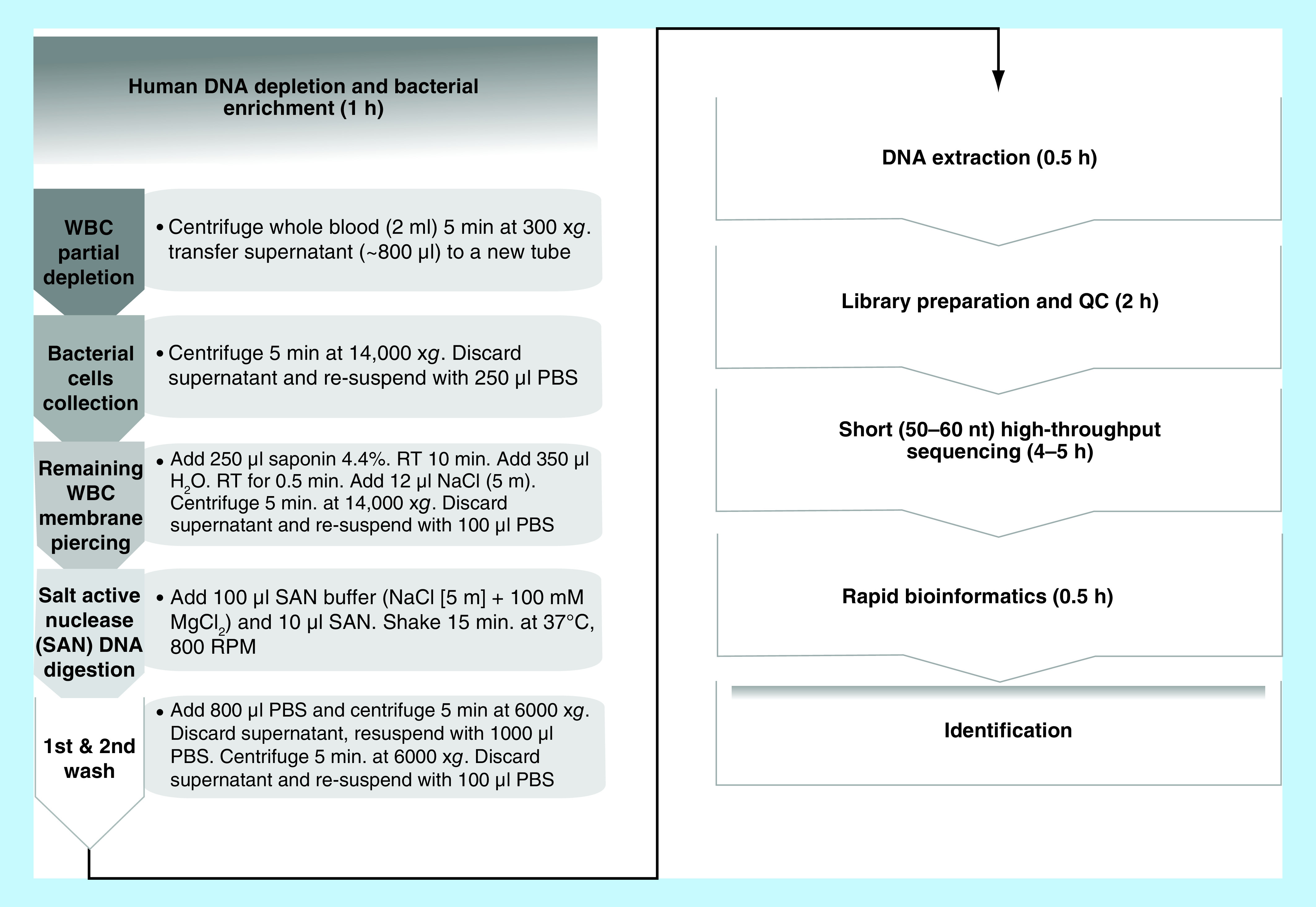
Flowchart illustrating the procedure of identification of pathogens in blood samples with unknown content with an emphasis on the depletion step. Depletion of white blood cells that carry most of the human DNA in a blood sample and elimination of the red blood cells which carry inhibiting levels of hemoglobin is carried out in our procedure. The human DNA depletion and bacterial enrichment step is elaborated in the gray polygons. On the right side of each polygon, there is a short description of the lab procedure and the rational is indicated on the left side. The following steps are elaborated in white boxes. The duration of each step (in h = h) is noted in brackets. nt: Nucleotide; PBS: Phosphate-buffered saline; QC: Quality control; WBC: White blood cell.

The approach described in this study for the rapid identification of pathogens in whole blood samples using HTS is summarized in Figure 1. We established a rapid and comprehensive procedure which starts with a whole blood sample of unknown content/suspicion and ends with a detailed metagenomic profile of the identified agents in a timeframe of ∼8 h.

The depletion process, which was partially adopted from Chralmpous *et al.* [16], with modifications suitable for whole blood samples, is provided in Figure 1. In our approach, the DNA is first treated in a depletion protocol, which is employed to enrich the pathogen/host ratio. Initially, using a differential centrifugation (300 ×*g*), the white blood cells, which contain most of the host DNA present in a whole blood sample, are removed (together with most of other types of blood cells), while most of the bacteria remain in the supernatant. This step lessens the possibility to detect obligatory intracellular bacteria using our approach. For the detection of such bacteria as well as viruses in clinical settings a different approach must be developed.

Next, we applied a saponin-based differential lysis method, which digests most of the remaining host DNA without any significant bacterial loss. This is achieved by the high concentrations of saponin (4.4%) and salt buffer (5.5M NaCl) that were found to be optimal for SAN digestion of DNA [16]. After the depletion step, the DNA is extracted and quantified utilizing the Qubit apparatus, which can detect sub-nanogram quantities of DNA. The HTS libraries are then processed using the NexteraXT technology. This technology simplifies the library preparation procedure, reduces the preparation time to less than 2 h and requires the smallest amount of DNA as a template compared with the requirements of other HTS library preparation procedures. The resulting libraries are sequenced, reads of 50–60 nt are generated and then taxonomically profiled against a comprehensive database of bacterial, viral and fungal genome sequences, using the PathoScope framework for rapid and accurate metagenomic profiling (Figure 1).

### Dual real-time PCR analysis of *Y. pestis* DNA enrichment & human DNA depletion in different conditions

**Figure 2.**
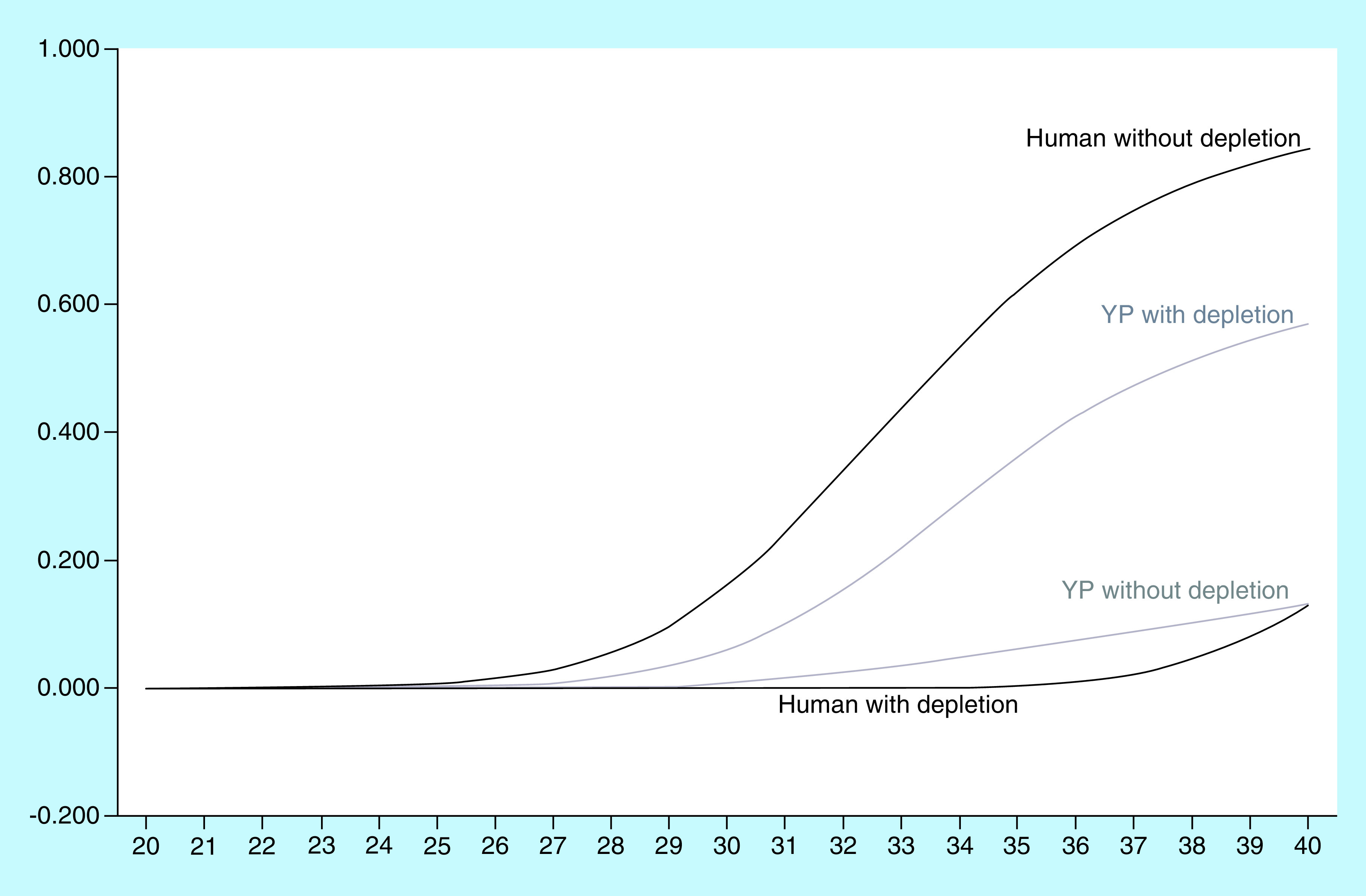
*Yersinia pestis* sample # 2 10^4^/ml before and after depletion. X axis: Ct. Y axis: ΔRN, the ratio of the fluorescence of FAM™ (YP) or VIC™ (human) dyes divided by the background fluorescence of ROX dye. YP: *Y. pestis*.

In order to examine the feasibility of our approach, we initially attempted to analyze whole blood samples with varying concentrations (10^3^–10^5^/ml) of spiked-in bacteria. We chose to spike-in *Y. pestis*, which represents, in our study, a typical Gram-negative bacterium. Two consecutive blood samples, freshly obtained from two volunteers, were spiked-in. To examine the efficiency of the depletion process, samples were analyzed by a dual RT-PCR, with specific tests for human and *Y. pestis* DNA, with (W) and without (WO) implementation of the depletion procedure. The results are summarized in Table 1. In all samples analyzed, a significant improvement in the host/pathogen DNA ratio was observed following the depletion process. For example, in sample 2, 10^4^ bacteria/ml spike-in, an improvement of 13.6 Ct was achieved, which corresponded to a 13,219-fold change and 4.1 orders of magnitude. Figure 2 illustrates the RT-PCR results, which show the vast decrease of the human DNA due to the depletion (from Ct 25.1 before the depletion process to Ct 35.6 after the process) and a less substantial elevation of the bacterial *Y. pestis* DNA (from Ct 30.0 before the depletion process to Ct 26.9 after the process).

**Table 1.** *Yersinia pestis* depletion process RT results.

WO/W	Conc. RT test/sample#	10^3^/ml				10^4^/ml				10^5^/ml			
		Ct	BΔ(Ct)	Total imp.		Ct	BΔ(Ct)	Total imp.		Ct	BΔ(Ct)	Total imp.	
			HΔ(Ct)	Ct	OM		HΔ(Ct)	Ct	OM		HΔ(Ct)	Ct	OM
**WO**	YP/1	40.0				31.0				27.2			
	Human/1	27.3				27.1				27.1			
**W**	YP/1	32.3	7.7	17.7	5.4	29.2	1.8	14.7	4.5	25.4	1.8	9.5	2.9
	Human/1	37.3	-10.0			40.0	-12.9			34.8	-7.7		
**WO**	YP/2	40.0				30.0				26.2			
	Human/2	26.4				25.1				25.5			
**W**	YP/2	29.1	10.9	21.4	6.5	26.9	3.1	13.6	4.1	22.9	3.2	12.5	3.8
	Human/2	36.9	-10.5			35.6	-10.5			34.8	-9.3		
**WO**	YP/2^†^	40.0				29.7				27.4			
	Human/2^†^	25.3				25.0				24.7			
**W**	YP/2^†^	31.0	9.1	19.5	5.9	27.2	2.5	17.5	5.3	22.9	4.5	16.9	5.1
	Human/2^†^	35.8	-10.5			40.0	-15.0			37.1	-12.5		
Average				19.5	5.9			15.3	4.6			13.0	3.9
Standard deviation				1.9	0.6			2.0	0.6			3.7	1.1

† = sample that was not freshly obtained.

WO: Without the depletion process. W: With the depletion process. YP: *Yersinia pestis* strain EV76. Conc.: Concentration (bacteria/ml). Ct: Critical threshold: the intersection between an amplification curve and the threshold line (see also in Figure 2). OM: Orders of magnitude. Total imp.: Total improvement of the host/pathogen ratio (in Ct or OM). BΔ: The subtractions of the Ct values between YP tests without and with the process. HΔ: The subtractions of the Ct values between human tests without and with the process.

To examine whether the depletion protocol can be utilized on samples that are not freshly obtained (e.g., samples that are not delivered directly from the patient to the lab), we spiked-in blood sample #2 with 10^3^ to 10^5^/ml of *Y. pestis* after 1 week at 4°C. As seen in Table 1, the improvement in the host/pathogen DNA ratio was similar to that obtained with fresh samples (see in the table sample2* compared with sample2 results).

The average improvement gained for the three samples analyzed was 19.5 (±8.4) Ct, which corresponds to 5.9 (±0.6) orders of magnitude; 15.3 (±2.0) Ct, 4.6 (±0.6) orders of magnitude and 13.0 (±3.7) Ct, 3.9 (±1.1) orders of magnitude, in 10^5^, 10^4^ and 10^3^/ml spiked-in bacteria, respectively. This improvement could be achieved primarily by the vast depletion of the host DNA. In addition, a minor elevation in the concentration of the bacterial DNA is obtained simply by the fact that the starting blood volume for the depletion procedure is 2 ml, while the final elution volume is 100 μl. Our data show that the concentration of the bacterial DNA is not hampered but increases utilizing the depletion procedure, while the human DNA is depleted in a very intensive manner, even till its level is completely undetectable by real-time PCR.

### Dual real-time PCR analysis of *B. anthracis* or *F. tularensis* enrichment & human DNA depletion

Following the results obtained for a typical Gram-negative bacterium, we wanted to check our approach using a serial dilution (10^3^–10^5^/ml) of spiked-in of *B. anthracis* or *F. tularensis*, which represent a typical Gram-positive bacterium and a typical facultative intracellular bacterium, respectively. Two consecutive blood samples, freshly obtained, were spiked-in with *B. anthracis* and *F. tularensis*. The samples were analyzed by a dual RT-PCR test with (W) and without (WO) the depletion process. The results are summarized in Table 2. Our results demonstrate that using these two spiked-in bacteria, there were significant improvements in the host/pathogen DNA ratio, following the depletion process in all the concentrations analyzed: in the case of the *F. tularensis* sample, the improvements were 10.1 Ct, which corresponds to 3.1 orders of magnitude; 15.6 Ct, 4.7 orders of magnitude and Ct 15.2, 4.6 orders of magnitude, in 10^5^, 10^4^ and 10^3^/ml spiked-in bacteria, respectively. For *B. anthracis*, the improvements were slightly lower, but still in all the concentrations tested, the improvement was ≥2.5-fold: 13.3 Ct, which corresponds to 4.0 orders of magnitude; 8.1 Ct, 2.5 orders of magnitude and 8.2, 2.5 orders of magnitude, in 10^5^, 10^4^ and 10^3^/ml spiked-in bacteria, respectively.

**Table 2.** *B. anthracis* and *F. tularensis* depletion process RT results.

WO/W	Conc. RT test	10^3^/ml				10^4^/ml				10^5^/ml			
		Ct	BΔ(Ct)	Total imp.		Ct	BΔ(Ct)	Total imp.		Ct	BΔ(Ct)	Total imp.	
			HΔ(Ct)	Ct	OM		HΔ(Ct)	Ct	OM		HΔ(Ct)	Ct	OM
**WO**	BA	35.7				29.9				27.6			
	Human	26.2				25.6				27.4			
**W**	BA	30.8	4.8	13.3	4.0	30.4	-0.6	8.1	2.5	28.3	-0.7	8.2	2.5
	Human	34.7	-8.4			34.2	-8.7			36.2	-8.8		
**WO**	FT	40				37.4				31.6			
	Human	29.5				28.1				28.2			
**W**	FT	38.8	1.2	10.1	3.1	32.0	5.4	15.6	4.7	28.2	3.4	15.2	4.6
	Human	38.5	-8.9			38.3	-10.2			40.0	-11.8		

WO: Without the depletion process. W: With the depletion process. Conc.: Concentration (bacteria/ml). Ct: Critical threshold: the intersection between an amplification curve and the threshold line (see also in Figure 2). BA: *B. anthracis* strain Vollum. FT: *F. tularensis* LVS. OM: Orders of magnitude. Total imp.: Total improvement of the host/pathogen ratio. (In Ct or OM). BΔ: The subtractions of the Ct values between BA or FT tests without and with the process. HΔ: The subtractions of the Ct values between human tests without and with the process.

### HTS of whole blood spiked-in depleted DNA samples

Finally, blood samples spiked-in with the three different bacteria used in this study (*B. anthracis, F. tularensis* and *Y. pestis*) in 10^3^–10^5^/ml concentrations were analyzed by our approach from sample to answer by HTS, in a working day. The *B. anthracis* and *Y. pestis* spiked-in samples were ran in a uniplex mode, while the* F. tularensis* samples were ran in a multiplex mode, with the three spiked-in concentrations multiplexed together. Using our pipeline we were able to diagnose *Y. pestis* in the corresponding spiked-in samples in all concentration tested (10^3^–10^5^/ml). For the *B. anthracis and F. tularensis* spiked-in samples, we were able to detect the corresponding spiked-in samples in the two higher concentrations (10^4^ and 10^5^/ml), but not in the lower concentration of 10^3^/ml. The strains that gained the highest number of hits and therefor identified by PathoScope are as follows: *Yersinia pestis CO92* for the *Y. pestis* spiked-in samples, *Bacillus anthracis* str. CDC 684 for *B. anthracis* and *Francisella tularensis* subsp. *holarctica* LVS for *F. tularensis* spiked-in samples (Table 3).

**Table 3.** HTS results summary.

Bacteria spike-in: concentrations	Positive specific reads^‡^	Total reads	Positive reads per million
YP: 10^5^/ml	22,212	8,396,771	2645
YP: 10^4^/ml	5252	17,471,984	301
YP: 10^3^/ml	655	10,344,988	63
BA: 10^5^/ml	3447	20,799,540	166
BA: 10^4^/ml	519	15,747,542	33
BA: 10^3^/ml	–	11,741,665	–
FT: 10^5^/ml^†^	2427	4,072,348	596
FT: 10^4^/ml^†^	215	5,367,217	40
FT: 10^3^/ml^†^	–	5,720,326	–

† = samples that were ran in a multiplex mode.

‡ The strains that were identified by PathoScope are as follows: *Yersinia pestis* CO92 for YP, *Bacillus anthracis str*. CDC 684 for BA and *Francisella tularensis* subsp. *holarctica* LVS for FT.

Spiked-in: YP: *Y. pestis* strain EV76. BA: *B. anthracis* strain Vollum ΔpXO1ΔpXO2 (ATCC 14578). FT: *F. tularensis* LVS.

The results presented in this paper demonstrate that our HTS-based approach has a promising potential for coping with the challenge of deciphering the composition of bacteremic whole blood samples without any prior knowledge about their nature or content. Using our approach, within a very applicable timeframe of ∼8 h, we succeeded to correctly identify three bio-threat agents, that acted as a proof-of-concept simulants, with a feasibly relevant limit of detection of 10^3^/ml–10^4^/ml spiked-in bacteria, in whole blood samples.

## Conclusion

Bacteremia and sepsis are significant conditions with high mortality and related costs. Unfortunately, routine blood cultures are too slow to aid swift therapeutic interventions. In this paper, we present a rapid and comprehensive procedure that starts with a whole blood sample of unknown content and ends with the generation of a detailed metagenomic profile of the identified bacteremic agents, in a timeframe of ∼8 h. Our approach, as a whole, is well suited for rapidly and unambiguously detecting any bacterial agent in whole blood samples, without any prior knowledge about the content of the sample. Our protocol could be used with other sequencing platforms, which might be faster, cheaper or simpler to use, for example, Oxford Nanopore. At this point in time, we did not test our approach using an obligatory intracellular bacterium, because a spike-in strategy is less relevant in this case. Moreover, our method currently is not suitable for detecting viruses in clinical settings. Our next goal is to further generalize the approach and to develop a holistic procedure that will detect any bacteria or virus in a whole blood sample.

## Future perspective

Using our approach and similar strategies for the depletion of host DNA, we anticipate that cases of bacteremia or even more minor infections will be diagnosed swiftly in a matter of minutes, using new and robust DNA sequencing machines that will be deployed near the patient’s bed-side. This diagnosis will be affordable and will include not only the detection of the illness-causing agent, without the need for any prior knowledge about its nature, but also traits such as possible antibiotic resistance and will facilitate therapeutic strategies.

**Summary points**

- Bacteremia and sepsis are significant conditions with high mortality and related costs. Routine blood cultures are too slow to aid swift therapeutic interventions. In the case of an indication for a specific pathogen, molecular detection methods could be applied directly from whole blood.
- When there is no previous information about the content of a sample, the detection method should be unbiased and based on high-throughput DNA sequencing (HTS). Direct diagnosis of whole blood samples utilizing HTS is very challenging, due to very high host/pathogen nucleic acids ratio.
- Varying concentrations of *B. anthracis, F. tularensis* and *Y. pestis, as simulants*, were spiked-in to whole blood samples and a novel human DNA depletion and bacterial enrichment procedure was applied followed by rapid HTS and bioinformatic analyses.
- Additionally, we performed dual (bacterial and human specific) RT-PCR analyses for each sample. Following the enrichment procedure, The RT-PCR results for *Y. pestis* spiked-in samples displayed an average improvement in the host/pathogen ratio of 5.9, 4.6 and 3.9 orders of magnitude (OM), in 10^5^, 10^4^ and 10^3^/ml spiked-in bacteria, respectively. *F. tularensis and B. anthracis* spiked-in samples demonstrated also a vast improvement, in the host/pathogen ratio, ranging from 3.1 to 4.7 OM for *F. tularensis* and 2.5 to 4.0 OM for *B. anthracis*.
- Using HTS, we were able to detect the corresponding spiked-in samples in concentration as low as 10^3^/ml for *Y. pestis* and 10^4^/ml for *B. anthracis and F. tularensis*. In this study, we established a comprehensive procedure that starts with an unknown blood sample and ends with a detailed metagenomic profile of the identified agents, in a timeframe of 8–9 h. Our approach has a promising potential for coping with the challenge of deciphering the composition of bacteremic whole blood samples without any prior knowledge about their nature, in a very relevant time frame.
